# Clinical practice guideline for the treatment of malignant ascites: section summary in Clinical Practice Guideline for peritoneal dissemination (2021)

**DOI:** 10.1007/s10147-021-02077-6

**Published:** 2021-11-20

**Authors:** Keisuke Matsusaki, Kuniaki Aridome, Shigenobu Emoto, Hiroaki Kajiyama, Nobumasa Takagaki, Takao Takahashi, Hiroshi Tsubamoto, Shoji Nagao, Akihiro Watanabe, Hideaki Shimada, Joji Kitayama

**Affiliations:** 1 Ascites Treatment Center, Kanamecho Hospital, Tokyo, Japan; 2 Department of Surgery, Saiseikai Sendai Hospital, Satsumasendai, Japan; 3 Department of Surgery Graduate School of Medicine, The University of Tokyo, Tokyo, Japan; 4 Department of Gynecology, Nagoya University, Nagoya, Japan; 5 Nobumasa Clinic, Kyoto, Japan; 6 Department of Supportive Oncology and Palliative Medicine, Saitama Medical University International Medical Center, Hidaka, Japan; 7 Department of Gynecology, Hyogo Medical University, Nishinomiya, Japan; 8 Department of Gynecology, Okayama University, Okayama, Japan; 9 Chikushi Nakagawa Hospital, Nakagawa, Japan; 10 Department of Gastroenterological Surgery and Clinical Oncology, Graduate School of Medicine, Toho University, Tokyo, Japan; 11 Department of Surgery, Jichi Medical University, Yakushiji 3311-1, Shimotsuke, 329-0498 Japan

**Keywords:** Malignant ascites, Peritoneal dissemination, Guideline, Peritoneovenous shunting, Cell-free and concentrated reinfusion therapy

## Abstract

Patients with peritoneal dissemination (PD) caused by abdominal malignancies are often associated with massive ascites, which shows extremely dismal prognosis because of the discontinuation of systemic chemotherapy mostly due to poor performance status. Many treatment methods, such as simple drainage, peritoneovenous shunting (PVS) and cell-free and concentrated reinfusion therapy (CART), have been used for symptom relief. However, the clinical efficacies of these methods have not been fully investigated yet. Recently, we developed the Clinical Practice Guideline for PD caused by various malignancies according to "Minds Clinical Practice Guideline Development Guide 2017". In this guideline, we systematically reviewed information on clinical diagnosis and treatments for PD using PubMed databases (2000 – 2020), and clarified the degree of recommendation for clinical questions (CQ). The evidence level was divided into groups by study design and quality. The literature level and a body of evidence were evaluated in reference to the Grading of Recommendations, Assessment, Development and Evaluation (GRADE) system. Based on the results of systematic review, the strength of the recommendations was evaluated at a consensus meeting of the Guideline Committee. This is the English synopsis of the part of treatment of malignant ascites in Clinical Practice Guideline for PD, 2021 in Japanese. The guidelines summarize the general aspect of the treatment of malignant ascites and statements with recommendation strengths, evidence levels, agreement rates and future perspective for four raised clinical questions.

## Introduction

Peritoneal dissemination (PD) frequently occurs in recurrent abdominal malignancy, such as gastrointestinal and ovarian cancer. In addition to systemic chemotherapy, many treatment methods have been developed to improve the outcome of the patients with PD. However, treatment selection largely varies among countries and institutions as well as types of cancer, which may make it difficult to objectively evaluate their therapeutic efficacy. The treatment for PD from various primary tumors has been mentioned separately in guidelines of each cancer type. However, there are relatively few descriptions about PD. In particular, massive ascites is commonly associated with PD caused by various malignancies with extremely poor prognosis. However, no standard treatment strategy has been established for patients with malignant ascites. Therefore, it is meaningful to present therapeutic guidelines specific to PD form cross-organ perspective.

In Clinical Practice Guideline for peritoneal dissemination (2021), information on various treatment for PD was summarized and the degree of recommendation for clinical questions (CQ) was clarified to produce a good social environment where medical professionals and patients well understand the outline of the treatment of PD and provide and enjoy high-quality medical care. Here, in this report, we show the summary of the section of malignant ascites in this Guideline.

## Methods

This guideline was basically created according to "Minds Clinical Practice Guideline Development Guide 2017" [1]. According to the primary cancer, it was divided to six subsections dealing with gastric cancer, pancreatic cancer, colon cancer, peritoneal pseudo-myxoma, ovarian cancer, and malignant ascites caused by all cancer types. In each section, themes that are difficult to judge in daily medical care were taken up as CQ. Systematic review was performed with related keywords for each CQ, and relating papers were comprehensively collected basically using PubMed databases (2000 – 2020). For some CQs with a small number of hit articles, additional papers in Igaku Chuo Zasshi (ICHUSHI), a Japanese bibliographic database, as well as Proceeding of annual meeting of American Society of Clinical Oncology (ASCO) were selected by hand search. The evidence level indicated by individual papers relating to the critical outcomes included within the CQs was divided into groups by study design and quality. The literature level and a body of evidence were evaluated in reference to the GRADE System and finally classified into four levels: "strong", "medium", "weak", and "very weak". Based on the results, draft recommendation statements and the strength of the recommendations were evaluated at a consensus meeting of the Guideline Committee. In discussion, the balance between the benefits and harms, patients’ values and hopes, cost effectiveness, and whether or not it can be performed at general facilities nationwide were taken into consideration. Finally, the strength of the recommendation was decided by a vote of committee members based on the GRADE Grid method. We selected one of the following five options in vote and recommendation was determined as follows.

(1) Strong “For” intervention, (2) Weak “For” intervention, (3) Weak “Against” intervention, (4) Strong “Against” intervention, (5) Not graded. With one vote, if 70% or more of the votes were obtained in any of (1) to (5), it was considered a final decision. If (1) + (2) exceeds 50% and (3) + (4) is 20% or lower, “weakly recommend to perform.” If (3) + (4) exceeds 50%, (1) + (2) is 20% or lower, “weakly recommend not to perform.” If this criterion cannot be met, then the results was disclosed and discussed and re-voted. If no agreement was reached again” (5) Not graded” was selected.

## Results and discussion

### General aspect of the treatment of malignant ascites

Massive ascites associated with cancerous peritonitis not only causes severe abdominal bloating and respiratory distress in the patient, but also reduces the patient's QOL and motivation to fight illness, which often leads to discontinuation of anti-cancer treatment. Guidelines for symptomatology relief for malignant ascites have already been published by the Japanese Society of Palliative Medicine in "Guidelines for Alleviation of Gastrointestinal Symptoms of Cancer Patients 2017 Edition" [2], which summarized the results of various studies on palliative therapies for ascites caused by PD, such as drug therapy, non-pharmacotherapy, and nursing care. However, in the case of large amounts of ascites, they rarely lead to effective anti-cancer treatments. Here, in this guideline, we defined “massive ascites” as ascites continuously accumulated from the pelvis to the sub diaphragm on CT images, and investigated the clinical efficacy of these therapeutic methods from the view point of the treatment of PD.

#### (1) Simple drainage

It can be safely performed with a small amount of 1–3 L according to the guidelines of the Japanese Society of Palliative Medicine and catheter placement is proposed when puncture occurs frequently [2]. Despite of its simple procedure, a prompt and temporary symptomatological relief can be usually obtained. However, in cases of large ascites retention, small drainage not only has a poor symptom-relieving effect, but also causes re-storage in a short period of time. Furthermore, there is concern that even a small amount of drainage may lead to deterioration of nutritional status, and that a large amount of drainage may lead to acute circulatory failure or renal failure. However, since it can be easily performed at any facility, it can be considered as the first-line treatment for massive ascites.

#### (2) Peritoneovenous shunting (PVS)

It was initially conceived in 1962 as a treatment that diverted the Holter valve for hydrocephalus, and then Le Veen shunt was developed with a movable silicon valve that opens and closes mechanically by increasing or decreasing the ascites pressure was devised in 1974, but they have not been widespread. After that, a Denver shunt was devised in 1990 that can manually pump ascites by a pump chamber with a check valve [3], which was widely used in various countries including Japan. Currently, there is no other useful equivalent. Although many reports on PVSs have been published, most of them are small-scale studies with less than dozens of cases. According to a relatively large multicenter study of 133 cases, the symptom relief rate was 83%, the time to onset of effect was 2 days (1–9 days), and the duration of symptom relief was 26 days (maximum 330 days). Adverse events occur in 6.8% of the patients, including bleeding, fever, thrombus, disseminated intravascular coagulation (DIC), pleural effusion, sepsis, intestinal obstruction, heart failure, with a high lethality rate of 4.5% [4]. Since the whole ascites enter from the superior vena cava to the systemic circulation, it is essential to anticipate serious complications, such as infection, abnormal coagulation, thrombosis, cardio-renal failure, severe lung injury as well as disperse of cancer cells throughout the body. Therefore, it cannot be used for mucous, purulent and biliary ascites and concentrated chyle. PVS is technically impossible ascites separated by multiple septal wall. In addition, shunt troubles include shunt obstruction, catheter rupture, and deviation. In particular, shunt obstruction is a frequent complication that occurs in about 16–45% after PVS placement [5, 6]. Causes of shunt occlusion include shunt lumen obstruction due to thrombosis, intra-abdominal fat, fibrin clot, catheter kink, fibrin sheath formation around the intravenous catheter, and encapsulation by the omentum around the intra-abdominal catheter [7]. Because of the technically difficult procedures and serious complications, currently less than 1000 cases are annually performed in Japan.

#### (3) Cell-free and concentrated ascites reinfusion therapy (CART)

**Fig. 1:**
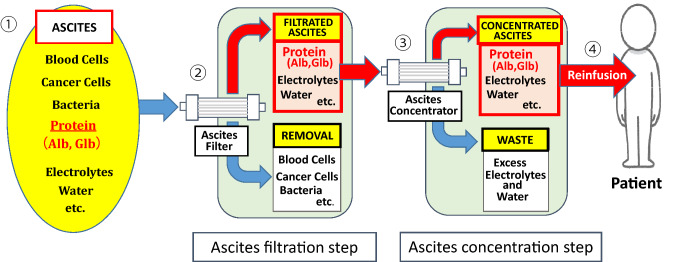
Schematic diagram of cell-free and concentrated ascites reinfusion therapy (CART). ① Total drainage of ascitic fluid from patient ② Cellular components of blood cells, cancer cells, bacteria, etc. removed from ascitic fluid by an ascitic fluid filter. ③ Excess water and electrolytes are removed by an ascitic fluid concentrator. ④ Concentrated protein solution is prepared and given to the patient by dripped infusion from peripheral vein

It was reported in 1973 as a filtration and concentration method using two types of Hollow Fibers for cancerous ascites [8] which is the prototype of CART. The current CART system was developed in 1977 and was approved by Japanese insurance system in 1981 (K-635). The schematic diagram of CART is shown in Fig. 1. However, since it is diverted from the dialysis system, there are many problems in ascites treatment, especially in case of cancerous ascites. First, the circuit and operation are complicated, and a skilled engineer and an expensive pump device for pressure control are required, and it takes time for priming and filtration concentration processing. In cancerous ascites, cell components, such as cancer cells and blood cells, as well as mucus or fibrin components often block the membrane pores after a few liter of ascites infiltration, making further treatment impossible. Moreover, mechanical stress and cell damage caused by excessive pressurization by a roller pump may lead to excessive production of toxic substances, such as inflammatory cytokines, endotoxins and high molecular weight mucus. Those soluble factors are also filtered and concentrated, causing serious side effects, such as high fever and septic shock.

Based on the above concerns, CART was considered to be dangerous to treat cancerous ascites, and was no longer used in the field of cancer treatment in the 1990s. Therefore, in clinical practice, CART was recognized as "a treatment with frequent side effects and poor therapeutic effect", and only used for the treatment for a small amount of hepatic ascites containing almost no membrane obstructive substance at some facilities in Japan without spreading overseas.

**Fig. 2:**
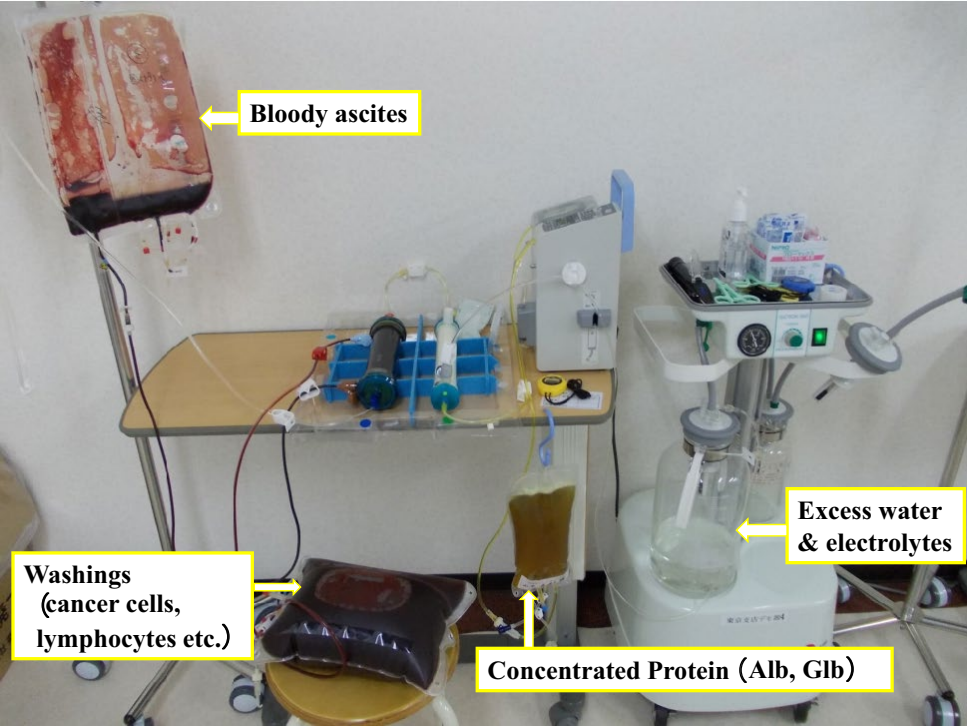
A case of KM-CART for bloody ascites (7.5 L) from a patient with peritoneal dissemination from gastric cancer

In 2008, Matsusaki developed an improved CART system that solves the conventional problems and a circulation management technique (KM-CART) for safe total drainage [9, 10]. The external pressure/constant pressure filtration method that does not stress the cellular components of ascites makes the treatment speed overwhelmingly fast at 3–5 min per liter (Fig. 2). Furthermore, the backwash function for filtration membrane obstruction has made it possible to treat a large amount of cancerous ascites of 10 L or more (maximum 28 L), and a large amount of cancer cells that can be recovered from the lavage fluid are being used for cancer research and treatment [11–13].

In recent years, CART has been actively used in combination with chemotherapy for peritoneal dissemination cases with a large amount of ascites [14–17]. When chemotherapy and CART are used in combination, there are concerns about side effects due to the concentration of the drug in ascites and reinfusion into the blood. Drugs bound to proteins, mainly albumin, cannot express anti-tumor activity and are neither metabolized nor excreted. Since the bound type drugs and the unbound free-type drugs maintain an equilibrium state with a certain binding constant, the free-type drugs can be supplied from the bound type, which may somehow affect the pharmacokinetics. However, albumin concentration in the ascites is generally lower than in the blood, and thus little clinical problem is expected unless CART is performed immediately after administration of the anticancer drug.

Some anticancer agents, such as Oxaliplatin, Docetaxel, and Paclitaxel, show a high binding rate to plasma proteins such as albumin [18, 19]. Therefore, when used in combination with intraperitoneal chemotherapy, it is expected that the total amount of drainage and intraperitoneal administration after CART will not only maintain the drug concentration but also enhance the antitumor effect by increasing the free form, which results in the excellent prognosis of patients with malignant ascites [15]. Since CART is easier to operate and has fewer complications compared to PVS, there are many facilities to perform it, and the number of cases is rapidly increasing to more than 40,000 cases per year in Japan. Furthermore, unlike PVS, cancer cells, mucus, cytokines, etc. are reduced from the abdominal cavity without spraying cancer cells in the blood. In the future, CART may become an indispensable supportive therapy in chemotherapy for patients with PD with large amounts of ascites. It is strongly desired to establish the good evidence by well-organized clinical trials.

## Clinical questions and recommendations

### CQ1: Is aggressive chemotherapy recommended for peritoneal dissemination with massive ascites?

Statement: According to the primary tumor, recommendation was decided as follows.

#### CQ1-1 Ovarian cancer

Ovarian cancer is extremely sensitive to chemotherapy. Although most patients with advanced PD have a large amount of ascites, long-term survival can be expected even from this condition. Chemotherapy is strongly recommended.

[Strength of recommendation; Strong, Strength of evidence; A, Consensus rate 100% (10/10)].

#### CQ1-2 Gastric cancer

In gastric cancer with PD with massive ascites, there are multiple retrospective studies suggesting that fluoropyrimidine-based chemotherapy prolongs progression-free survival and overall survival. Chemotherapy is recommended after careful evaluation of general condition of patients.

[Strength of recommendation; Weak, Strength of evidence; C, Consensus rate 100% (10/10)].

#### CQ1-3 Other cancers

There are few data supporting the usefulness of chemotherapy for massive ascites in carcinomas other than ovarian and gastric cancers. Chemotherapy is generally not recommended except special cases. Indications and regimen selection should be carefully considered for each individual patient.

[Not recommended; Strength of evidence; D Consensus rate 70% (7/10)].

Recommendations for tomorrow: Chemotherapy is standard treatment for patients with PD with massive ascites in ovarian cancer, whereas strong evidence has not been established for other cancers. Due to poor performance status (PS) and difficulty in oral intake, it is often excluded in clinical trials. In some clinical trials, patients with PS2 and poor oral intake enrolled, which suggests if chemotherapy reduces the volume of ascites, their prognosis can be prolonged. Clinical trials focused on the patients with massive ascites to evaluate the real efficacy of chemotherapy with various treatment regimens including intraperitoneal administration as well as the combination with CART is desired.

### CQ2 Is CART recommended for patients with peritoneal dissemination with massive ascites?

Statement: CART is effective in improving symptoms, such as abdominal bloating and loss of appetite. It can be safely performed and leads to saving of blood products. It is strongly recommended to perform CART for patients with massive cancerous ascites.

[Strength of recommendation; Weak, Strength of evidence; C, Consensus rate 100% (10/10)].

Recommendations for tomorrow: Relieving distress for patients with massive ascites is a clinically crucial issue. Clinical studies are desired to verify the usefulness of CART for the treatment of cancerous ascites.

### CQ3: Is abdominal-venous shunting recommended for massive ascites with peritoneal dissemination?

Statement: Patients with massive ascites with PD often have a predicted life expectancy of PS3 on a weekly basis, and abdominal bloating and repeated abdominal punctures can be avoided by performing abdominal-venous shunt (AVS, Denver's shunt). However, the complications are frequent and can be fatal and thus abdominal-venous shunting is strongly recommended not to be performed.

[Strength of recommendation; Weak, Strength of evidence; C, Consensus rate 70% (7/10)].

### CQ4 Is intraperitoneal administration of triamcinolone acetonide recommended for cancerous ascites control?

Statement: It is a treatment option that is easy to operate and accessible to patients, but no clear recommendations can be made.

[Not recommended; Strength of evidence; D, Consensus rate 100% (10/10)].

Recommendations for tomorrow: Since it is difficult to conduct controlled trials in end-of-life care, off-label use is desirable with sufficient informed consent for complications.
